# Peritoneal Dialysis in Patients Supported by Left Ventricular Assist Device

**DOI:** 10.1111/aor.14970

**Published:** 2025-02-26

**Authors:** Zurab Darbaidze, Bastian Schmack, Günes Dogan, Ali Saad Merzah, Maria M. Gabriel, Adelheid Görler, Aron‐Frederik Popov, Alexander Weymann, Arjang Ruhparwar, Jan D. Schmitto, Jasmin S. Hanke

**Affiliations:** ^1^ Department of Cardiothoracic, Transplantation and Vascular Surgery Hannover Medical School Hannover Germany; ^2^ Department of Neurology Hannover Medical School Hannover Germany

**Keywords:** APD, CAPD, cardiorenal syndrome, continuous ambulatory peritoneal dialysis, heart failure, kidney failure, left ventricular assist device, LVAD, peritoneal dialysis

## Abstract

**Introduction:**

Terminal heart failure is often associated with end‐stage kidney disease. Due to advantages concerning patient independence, peritoneal dialysis (PD) is an alternative to conventional hemodialysis treatment. As left ventricular assist device implantations continuously increase, data on combined PD and LVAD is rare. We present the first and largest cohort study on this exclusive patient cohort.

**Methods:**

A retrospective study was conducted on patients who underwent LVAD implantation at a high‐volume heart failure center from 2000 to 2024. Adverse events were analyzed according to the INTERMACS classification.

**Results:**

A total of nine patients were identified as undergoing PD on LVAD therapy. Mean age at the time of LVAD implantation was 67 years. Main cause of kidney disease was cardio‐renal syndrome (67%). In all patients, PD therapy was established before LVAD implantation. Mean time on PD before LVAD implantation was 72 months. None of the patients were weaned from dialysis nor were converted to conventional dialysis. Four patients experienced driveline infection. Three patients suffered an infection of their PD catheter. A combination of PD and DL infection was detected in two cases. None of these infections were associated with the same pathogens. Mean survival after LVAD + PD was 56.5 months.

**Conclusion:**

Peritoneal dialysis has advantages over hemodialysis including fewer bloodstream infections, fewer hemodynamic shifts, and the comfort of the ambulant setting. This study illustrates that PD in LVAD patients is feasible and long‐term support up to several years is achievable without major complications.

## Graphical abstract

Peritoneal Dialysis (PD) has advantages over hemodialysis, including fewer infections and the comfort of the ambulant setting. This study illustrates that PD in LVAD patients is feasible and long‐term support up to several years is achievable without significant complications.

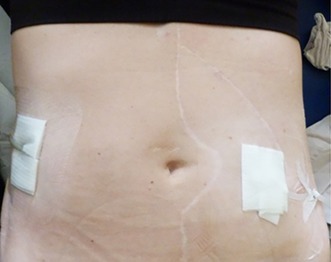

## 1 Introduction

Left ventricular assist device (LVAD) implantation is a well‐established treatment option in end‐stage heart failure (HF). Late‐stage heart failure in LVAD candidates is often associated with renal failure, defined as cardiorenal syndrome (CRS), which describes the complex interaction between the two organ systems [1, 2, 3, 4, 5, 6, 7].

Cardiorenal syndrome represents the confluence of heart‐kidney interactions across several interfaces. These include the hemodynamic cross‐talk between the failing heart and the response of the kidneys and vice versa. CRS is associated with worsening of outcomes and complexities of diagnostic, preventive, and therapeutic approaches [1, 2, 3].

The only therapeutic treatment for terminal kidney disease is solid organ transplantation. However, due to donor scarcity and strict selection processes, the option of kidney or combined kidney and cardiac transplantation is often not available for LVAD patients.

Symptomatic treatment for terminal renal failure is the removal of toxins via dialysis. There are two forms of permanent dialysis treatments: Hemo‐ and Peritoneal Dialysis [4, 5, 6, 7, 8]. HD results in severe impairment in the quality of life (QoL) by a strict protocol of up to four visits to a dialysis center per week [1, 3, 5]. Contrarily, peritoneal dialysis (PD) allows patients to perform dialysis treatment at home independently. PD removes uremic toxins and fluid through the peritoneum by instillation and drainage of toxins via a PD catheter in and out of the abdominal cavity.

There are two Types of peritoneal dialysis: Continuous Ambulatory Peritoneal Dialysis (CAPD) and Automated Peritoneal Dialysis (APD). The main differences between the two types of peritoneal dialysis is the schedule of fluid exchanges and the use of an automatic machine versus a manual fluid exchange. CAPD, exchanges usually take place four times a day. A dialysate fluid is put through the patient's catheter by gravity, which stays in the abdomen for about 4 h. During this treatment, the patient can move and do activities as normal. After 4 h the dialysate fluid is drained out of the abdomen via the patient's catheter. In APD a machine automatically and continuously moves dialysate into and out of the abdomen, which usually takes place overnight [9].

The major advantage of PD is patient's independence from regular and lengthy visits to dialysis clinics which leads to increased rates of self‐efficiency and quality of life. Despite its increased flexibility for the patients, PD requires high patient's compliance to prevent complications such as infection, hypoalbuminemia and hypotension. The additional intraabdominal fluid may also lead to inguinal hernia [10, 11]. Bacterial peritonitis is the worst complication of PD, requires prompt diagnosis and treatment with antibiotics to prevent complications such as sepsis, shock, and multiple organ failure. Treatment usually involves intravenous antibiotics to target the specific bacteria causing the infection, along with supportive measures such as fluid resuscitation and pain management. In coherence with LVAD driveline infections, surgery may be necessary to repair the catheter insertion site due to infection [10].

**FIGURE 1:**
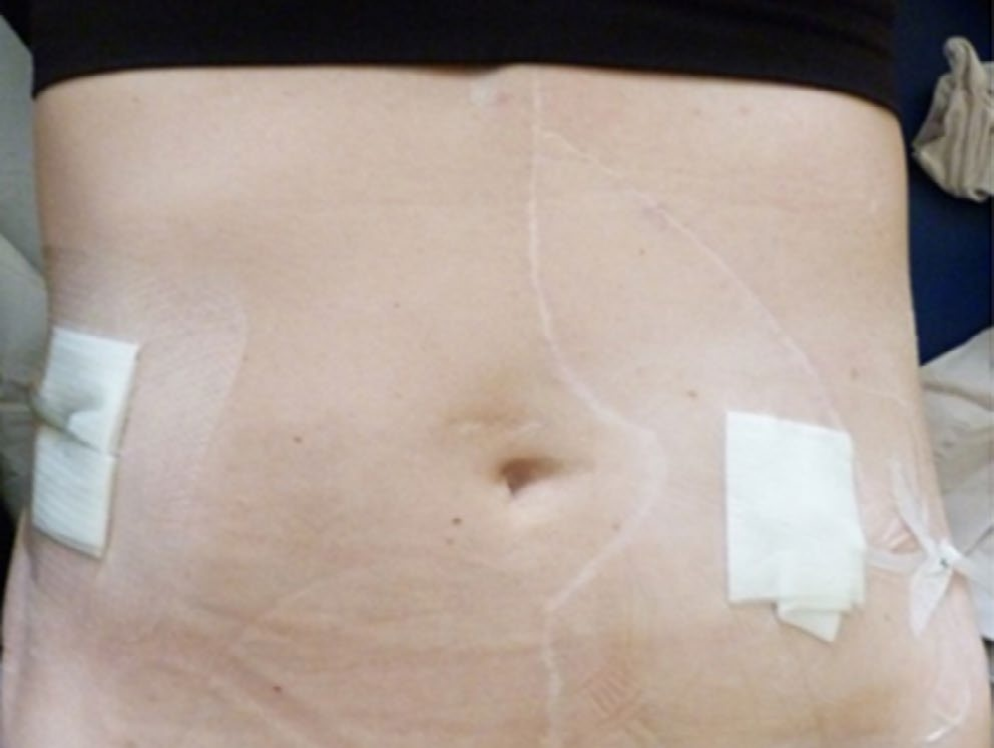
Combined therapy with peritoneal dialysis and left ventricular assist device. Figure displaying driveline exit site as well as peritoneal dialysis catheter. [Color figure can be viewed at wileyonlinelibrary.com]

According to the MOMENTUM three study about 20%–25% of LVAD patients require dialysis treatment before or after LVAD implantation [11]. Despite the benefits of PD, the majority of VAD patients receive conventional hemodialysis treatment. Theoretical adverse events of combined LVAD and PD therapy may include lack of compliance and volume overload resulting in right heart failure as well as an increased risk of driveline and PD catheter infection resulting in peritonitis [10, 12] (Figure 1). Nevertheless, potential benefits of PD in LVAD patients is the increased patient's independence resulting in improved QoL.

Due to the underutilization of therapy, data on PD in LVAD patients is rare [9, 12]. With this study, we report on the first cohort series of LVAD patients undergoing peritoneal dialysis treatment with a focus on outcomes and adverse events.

## 2 Material and Methods

A retrospective study was conducted on patients who underwent left ventricular assist device implantation at a high‐volume heart failure center from 2000 to 2024. Inclusion criterion was age > 18 years and combined therapy with peritoneal dialysis. Preoperatively, all patients gave written consent for LVAD implantation. If patients were unable to do so for medical reasons, informed consent was obtained from their relatives or guardians. Data was retrospectively acquired using in‐house clinical databases. Among baseline characteristics, adverse events and survival data were collected and analyzed. The investigation conforms to the principles outlined in the Declaration of Helsinki. Due to the retrospective nature of this study, the local institutional ethics committees waived the need for IRB consent for data collection and approved the study.

## 3 Statistical Analysis

Raw data were collected and prepared in a Microsoft Excel 2020 database (Microsoft Corporation, Redmond, USA), ensuring structured and accessible datasets for subsequent analysis. All statistical analyses were performed using Software SPSS 27 (*IBM SPSS Statistics Version 27*). Categorical variables are reported in frequencies and continuous variables are reported as mean ± standard deviation or as median with range if applicable. Adverse events were recorded and analyzed according to the standard INTERMACS classifications.

## 4 Results

More than 500 patients were screened for the study. Out of those, a total of nine patients were identified as undergoing peritoneal dialysis on LVAD therapy. Baseline characteristics of study cohort are displayed in Table 1. The majority of patients was male (7/9, 77%). Mean age at the time of LVAD implantation was 67 years (Range: 59 to 81 years). The most common cardiac diagnosis was dilated cardiomyopathy (7/9, 77%). Four patients were supported by HeartWare HVAD (HeartWare, Medtronic, USA) and 5 by HeartMate 3 (Abbott, U.S.A.). Main cause of chronic kidney disease was cardio‐renal syndrome (6/9, 67%). Other diagnoses included glomerulonephritis (2/9, 22%) and hydronephrosis (1/9, 11%). None of the patients was on extracorporeal mechanical support before or after LVAD implantation. In all patients PD therapy was established prior to LVAD implantation. Mean days on PD before LVAD implantation was 866 (72.1 months) (Range: 8 to 2951 days). The majority of patients utilized continuous ambulatory PD home dialysis (CAPD). Only one patient underwent PD via automated Perioneal Dialysis (APD). All patients received standard of care after LVAD implantation. No additional antibiotic therapy after the initial postoperative phase was necessary. Mean days on LVAD plus PD was 678.5 (56.5 months) (Range: 54 to 2585 days). None of the patients underwent consecutive kidney or heart transplantation and none of the patients was weaned from dialysis after LVAD implantation.

**TABLE 1:** Baseline characteristics of study cohort.

Baseline characteristics of the study cohort	
Study cohort (*n*)	*n* = 9
Female *n*, (%)	2 (23)
Male *n*, (%)	7 (77)
Average age years., (range)	68; (59–81)
Ischemic cardiomyopathy *n*, (%)	2 (23)
Dilated cardiomyopathy *n*, (%)	7 (77)
Type of LVAD	
HeartMate 3 *n*, (%)	4 (44)
HeartWare HVAD *n*, (%)	5 (56)
Main range postoperative ejection fraction after LVAD implantation	
LVEF (%)	15–30
RVEF (%)	30–50
Onset of peritoneal dialysis	
Pre‐LVAD PD *n* (%)	9 (100)
New PD Post LVAD‐Implantation *n*, (%)	0 (0)
Type of peritoneal dialysis	
CAPD *n*, (%)	8 (89)
APD *n*, (%)	1 (11)
Etiology of renal failure	
Cardiorenal syndrome *n*, (%)	6 (67)
Glomerulonephritis *n*, (%)	2 (23)
Hydronephrosis *n*, (%)	1 (11)
Mean months on PD *n*, range days	72.1; (8–2951)
Mean months on PD + LVAD *n*, range	56.5; (54–2585)

Abbreviations: APD, automated peritoneal dialysis; CAPD, continuous ambulatory peritoneal dialysis; LVAD, left ventricular assist device; LVEF, left ventricular ejection fraction; PD, peritoneal dialysis; RVEF, right ventricular ejection fraction.

Adverse events and outcomes are displayed in Table 2. In our cohort, four patients experienced driveline infection (44%, 4/9). Pathogens were *Staphylococcus epidermidis* and *Enterococcus faecium* (75%; 3/4 and 25%; 1/4). Three patients of the cohort (3/9, 33%) suffered an infection of their PD catheter. Out of those one patient experienced secondary peritonitis (1/3, 33%). In one case PD catheter infection was already present before LVAD implantation. In the other cases, the mean timespan on LVAD until infection of PD catheter was 650 days (range: 210–1047). A combination of PD and DL infection was detected in two cases (2/9; 22%). None of these infections was associated with the same pathogens. Treatment of PD catheter and DL infection was performed by antibiotic therapy. Surgical revision was not necessary in either case.

**TABLE 2:** Adverse events and outcomes of the study cohort.

Adverse events and outcomes	
Study cohort	*n* = 9
Driveline infection *n*, (%)	4 (44)
Major pathogen of DL‐infection *n*, (%)	
*Staphylococcus epidermidis*	2 (50)
*Escherichia coli*	1 (25)
*Staphylococcus haemolyticus*	1 (25)
PD Infection *n*, (%)	3 (33)
Major pathogen of PD infection *n*, (%)	
*Escherichia coli*	2 (66)
Enterococcus species	1 (33)
Peritonitis *n*, (%)	1 (11)
Pathogen of peritonitis *n*, (%)	
*Escherichia coli*	1 (100)
Mean Survival on PD + LVAD years (months); (range in days)	4.6 (56.5) (54–2585)
Causes of death *n*, (%), POD	
Multi‐organ failure	3; POD 54; 269; 2585
Neurologic complications	
Stroke	1 (11); POD 1261
ICB	1 (11); POD 113
SAB	1 (11); POD 1523
Sepsis with peritonitis	1 (11); POD 101
Right heart failure	1 (11); POD 78

Abbreviations: LVAD, Left ventricular assist device; PD, peritoneal dialysis.

PD failure was not detected in our cohort. None of the patients was converted to conventional dialysis treatment. Reasons for re‐admittance were cardiac decompensation due to right heart failure, Driveline infection with or without sepsis and infect triggered INR derailment.

In the long term follow up, right heart failure was diagnosed in two patients. One patient died in the postoperative course after LVAD implantation on POD 78 on intensive care unit due to biventricular failure with emphasis on terminal right heart failure. The other patient was diagnosed with right heart failure before LVAD implantation but was stabilized via fluid management und heart failure medication and was successfully discharged home with PD. This patient later died on POD 269 due to multiorgan failure.

Mean survival after LVAD + PD was 678.5 days (56.5 months) (Range: 54 to 2585 days). Causes of death included multiorgan failure (MOF) (3/9; 33%); neurological adverse events (3/9; 33%); sepsis with peritonitis (1/9. 11%) and right heart failure (1/9; 11%). One patient is still ongoing on the device (1/9; 11%).

## 5 Discussion

For combined LVAD and CAPD therapy, both systems' risk factors need to be considered. Common long term complications of LVAD therapy are hemocompatibility associated adverse events as well as right heart failure, which is associated with fluid management and device infections [13]. CAPD therapy is also prone to a number of early (< 30 days) and late (> 30 days) complications, including outflow failure and infections of the exit site or tunnel, as well as peritonitis [4]. Therefore, both therapeutic strategies require high patient compliance due to the self‐management of the specifics of either device [1].

Data on combined PD and LVAD therapy is rare. To our knowledge only case reports are published on this exceptional group of patients. In contrast to our cohort, the mentioned case reports describe patients, who received PD after LVAD implantation. Forcey et al. presented a case of an LVAD patient, who successfully was bridged for 10 months to a combined heart and kidney transplant via LVAD and PD [14]. Koppel et al. describe a case of a 55‐year‐old man, who received PD therapy after LVAD implantation and was supported by a combination of treatments for 16 months [6]. Guglielmi et al. as well as Ajuria et al. describe cases of LVAD patients, who were successfully treated by APD [13, 15]. In most cases, the decision to perform PD over conventional dialysis was based on experienced hypotension during traditional dialysis treatment, which led to consecutive LVAD alarms. Equally, increased patient independence influenced the decision for PD treatment. No interferences between PD catheter and driveline infections, nor cases of pertonitis were reported in all described cases. No case of right heart failure nor PD failure was described.

In our cohort of nine patients one patient suffered a severe bacterial peritonitis, which resulted in the death of the patient. However, the infection was not associated with an LVAD associated infection. In general the risk of peritonitis across all patient groups is described with 15% in literature [16].

Based on our experience, therapeutic success is determined by such parameters as structured outpatient clinic care, accurate anticoagulation therapy monitoring, diligent LVAD‐driveline and CAPD‐catheter care/stabilization, complete rehabilitation and a healthy lifestyle (regarding nutrition and exercise). Careful monitoring for adverse events and an adequate response plan are necessary for avoiding severe adverse events such as right heart failure, hemodynamic decompensation and device infection. Patient selection and compliance are also equally for ideal outcomes [6, 15, 16]. Exclusion criteria for PD are abdominal adhesions and chronic inflammatory abdominal disease.

A disadvantage of PD is the daily need for dialysis treatment. An alternative to PD is home hemodialysis, which enables patients to perform hemodialysis treatment mostly independently from dialysis clinics at home on their own schedule. A case report on an LVAD patient undergoing successful home dialysis has been reported by Hanna et al. [17]. However, home dialysis remains expensive and not available in many countries. Also, high patient compliance is required with the need for caution for low volume and suction events as well as a possible risk of bleeding or thrombosis due to the need for long‐term anticoagulation in LVAD patients [17]. Yet, home hemodialysis is becoming more popular and is feasible in highly selected patients.

Concluding, this case series shows that the combined therapy with CAPD and LVAD systems is feasible if the patient's safety is ensured by education, outpatient care, and diligent monitoring. In our cohort, up to 2858 days (95.2 months) of combined therapy were achieved.

## 6 Limitations

This study has limitations. Due to the nature of this study, the data is retrospectively collected and analyzed and therefore it is subject to the limitations associated with retrospective studies. Additionally, the number of patients was small and the study timeframe covered several years, which reduces the statistical power and ability to infer positive findings. Thus, the results of this analysis might be affected by the overall technical improvements of newer generation VADs. In addition, the results of practical studies are prone to learning curves and single center's specific characteristics. As such, larger studies are needed further to study the aspects of combined PD and LVAD therapy.

## 7 Conclusion

Due to cardiorenal syndrome, renal function often gradually decreases in heart failure patients and may require dialysis treatment. Hence, some patients receive peritoneal diaysis before VAD implantation. Conceptually, PD has advantages over hemodialysis, including fewer bloodstream infections, fewer hemodynamic shifts, and the comfort of the ambulant setting, which is especially relevant to destination therapy patients. This study illustrates that PD in LVAD patients is feasible and long‐term support up to several years is achievable without significant complications.

## Author Contributions

Z.D., conception and design; analysis and interpretation; writing the article, critical revision of the article; final approval of the article; data collection, provision of materials, patients, or resources; statistical expertise; literature search. B.S., conception and design; analysis and interpretation; writing the article, critical revision of the article; final approval of the article; data collection, patients, or resources; literature search; administrative, technical, or logistic support. G.D., data collection, technical and logistic support; provision of materials, patients, or resources; critical revision of the article; final approval of the article. A.S.M., statistical expertise, data preparation, evaluation and illustration, critical revision of the article. M.M.G., provision of materials, patients, or resources; critical revision of the article; final approval of the article. A.G., provision of materials, patients, or resources; critical revision of the article; final approval of the article. A.F.P., provision of materials, patients, or resources; critical revision of the article; final approval of the article. A.W., provision of materials, patients, or resources; critical revision of the article; final approval of the article. A.R., provision of materials, patients, or resources; critical revision of the article; final approval of the article. J.D.S., provision of materials, patients, or resources; critical revision of the article; final approval of the article. J.S.H., conception and design; analysis and interpretation; writing the article, critical revision of the article; final approval of the article; data collection, provision of materials, patients, or resources; statistical expertise; literature search.

## Disclosure

The authors have nothing to report.

## Conflicts of Interest

J.D.S. and G.D. are consultants for Abbott. The other authors declare no conflicts of interest.
